# The reproductive pattern and potential of free ranging female wild boars (*Sus scrofa*) in Sweden

**DOI:** 10.1186/s13028-017-0321-0

**Published:** 2017-08-01

**Authors:** Anna Malmsten, Gunnar Jansson, Nils Lundeheim, Anne-Marie Dalin

**Affiliations:** 1 Division of Reproduction, Department of Clinical Sciences, Swedish University of Agricultural Sciences, Box 7054, 750 07 Uppsala, Sweden; 2 Grimsö Wildlife Research Station, Department of Ecology, Swedish University of Agricultural Sciences, 730 91 Riddarhyttan, Sweden; 3 Department of Animal Breeding and Genetics, Swedish University of Agricultural Sciences, Box 7054, 750 07 Uppsala, Sweden

**Keywords:** Litter size, Ovulation rate, Pregnancy rate, Seasonality, Supplemental feeding

## Abstract

**Background:**

The number and spatial distribution of wild boars (*Sus scrofa*) has increased remarkably in Sweden as well as in other European countries. To understand the population dynamics of the wild boar, knowledge of its reproductive period, oestrus cycle and reproductive success is essential. The aim of this study was therefore to describe the seasonal reproductive pattern and reproductive potential of a wild boar population in Sweden. The study was based on findings from macroscopic examinations of the reproductive organs from 575 hunter-harvested female wild boars (>30 kg body weight). Samples were collected between December 2011 and December 2015 in the southern and middle parts of Sweden. The age of the sampled animals was determined and dressed weight was noted. The stage of the reproductive cycle was defined according to ovarian structures and in relation to the appearance of/and findings in the uterus. The crown-rump length (CRL) of the embryos/foetuses was used to calculate the oestrus/mating month and month for the expected farrowing.

**Results:**

The macroscopic examination revealed a seasonal variation of reproductive stages, although cyclic and pregnant females were found in all seasons. Moreover, the estimated oestrus/mating and farrowing months based on the CRL showed that mating and farrowing may occur ‘off-season’. The average litter size (no. of embryos or foetuses) per pregnant female was 5.4. Sow weight and age had significant effect on both the reproductive potential (ovulation rate and litter size) and pregnancy rate, respectively.

**Conclusions:**

The reproductive potential in the studied wild boar population was high compared to studies from other countries and farrowing may occur ‘off-season’. This suggests that the environmental conditions in Sweden, including supplemental feeding, are favourable for wild boar reproduction.

**Electronic supplementary material:**

The online version of this article (doi:10.1186/s13028-017-0321-0) contains supplementary material, which is available to authorized users.

## Background

The number and spatial distribution of wild boars has increased remarkably in Sweden as well as in other European countries during the last decades [1–5]. Besides the generalist behaviour of wild boars such as wide feed and habitat selection, the main reasons for this rapid increase are thought to be the high reproductive potential of the wild boar [6] in combination with extensive supplemental feeding and relatively low harvest rates.

To understand the population dynamics of a game species such as the wild boar, knowledge of its reproductive period, oestrus cycle and reproductive success is essential [7, 8]. Wild boar biology has been studied in several countries with a long history of wild boar populations. Based on European studies, pure breeds of wild boars without a history of hybridization with domestic pigs (that are poly-oestral throughout the year) have a reproduction that is clearly seasonal [9]. The wild boars are so-called short-day breeders. During the summer months, the majority of the female wild boars are anoestral [9–11]. In continental Europe, the summer anoestrus coincides with high ambient temperatures, long days and restricted access to feed [12]. In the autumn, as a result of shortened day length, the oestrus period begins [9]. However, the first oestrus of the breeding season may take place at different time in different years depending on natural feed availability and other environmental and climatic factors [9, 11, 13]. For example, it appears that earliness or delay in the onset of the breeding season is related to the level of available feed in that a high access to feed can cause a shortened anoestrus period [10]. In a natural environment, without any human impact, there is normally only one oestrus peak in the autumn/winter [12]. However, a bimodal pattern of oestrus and consequently farrowing can also occur [2, 14]. In addition, some studies show that farrowing may occur throughout the year when feed is available all year round [15, 16].

The current wild boar population in Sweden originates from an unknown number of individuals that escaped enclosures in the 1970s and 1980s. Since, the population has steadily and rapidly increased. During the last 20 years, the annual number of harvested wild boars in Sweden has increased from 5000 to 100,000 animals [5]. The knowledge about Swedish wild boar and its management is limited, and only a few studies have been published. These studies have mainly described wild boar movement patterns, behaviour, and habitat selection [17–20], but few include data on reproduction [7, 21], i.e. reproductive performance and reproductive potential of the species in Sweden.

The aim of this study was to describe the reproductive pattern and potential of free ranging female wild boars in Sweden.

## Methods

### Study area

Samples were collected between December 2011 and December 2015 at seven hunting estates in four different regions (Skåne, Blekinge, Södermanland, and Uppland) in southern and central Sweden (Additional file 1).

The size of the estates varied from 10 to 87 km^2^ and the population density of wild boars estimated by the wildlife manager at each estate ranged from 5 to 40 animals per km^2^ among the estates. Similar management strategies for the wild boars were applied at all estates, including supplemental feeding throughout the year. The types of feed used were corn, grain, mixes of cereal and sugar beets, and silage (anaerobic fermentation with wheat and oats grains, peas, and clover). Meteorological data (monthly mean precipitation and temperature) were obtained from local meteorological services [22]. During the study period, the weather conditions in the sampling regions were similar with mean temperatures in January and July of −1 and 18 °C respectively, and mean precipitation in January and July was 49 and 58 mm, respectively (Additional file 2).

### Hunting methods and Swedish wild boar hunting legislation

Sampling occurred during ordinary hunting. The hunting methods used were beat/drive hunts and stalking. Beat/drive hunts were the most common method in the study areas during the main hunting season (October until February) and resulted in considerable hunting bags (30–100 animals per day). Stalking was used during spring and summer and animals were most often shot while feeding at open field/grazing-ground, i.e. hunting was used mainly as a crop preventive action.

For practical reasons, the sampling effort was biased towards the main hunting season (October–February). In Sweden, the hunting season of adult wild boars range between 16th of April and 15th of February. The wild boar hunting is allowed 24 h, i.e. day and night, and hunting with dogs is allowed between 1st of August and 31st of January. Hunting of yearlings is allowed throughout the year, whereas females with dependent piglets are protected [23].

### Data collection

Body weight (BW) and field dressed weight (FDW, weight of the eviscerated animal with skin) were recorded. Sampling included all females with BW ≥30 kg. For animals where only BW was noted, FDW were estimated using the equation FDW = −1.855 + 0.810*BW, based on the known relationship between BW and FDW of 296 animals [Lundeheim, unpublished data]. Altogether, FDW was noted for 540 animals. Age was determined for 442 of the animals based on tooth eruption and replacement pattern [24] and the animals were subdivided into juveniles (<1 year), yearlings (1–2 years) and adults (>2 years).

### Macroscopic examinations of reproductive organs

The collected reproductive organs were frozen at −20 °C until the macroscopic laboratory examination [7]. The size and weight of the uterus and ovaries were recorded as were the ovarian structures (follicles and *corpora lutea,* CL). Ovulation rate (OR) was defined as the total number of active CL from both ovaries. The uteri were cut open and the contents examined. The reproductive stage was determined based on the data from the macroscopic examination [7]. Animals in prooestrus, oestrus, metoestrus and dioestrus were classified as cyclic. In pregnant females, from about 18 days of pregnancy (when embryos in the foetal membranes could be clearly observed), the number of embryos or foetuses were counted. Litter size was defined as the total number of embryos or foetuses. The OR and litter size were considered as two measurements of reproductive potential. The crown-rump length (CRL), weight, gender and development of the embryos/foetus were noted. The estimated oestrus/mating months and expected farrowing months were calculated based on the CRL of the embryos/foetuses [25].

### Statistical analyses

The statistical analyses were carried out in SAS (SAS Institute Inc. Cary, N. C). In the analyses, year was divided into four seasons as: spring (March–May), summer (June–August), autumn (September–November) and winter (December–February). Analysis of variance [both PROC GLM (general linear model) and PROC GLIMMIX (generalized linear mixed model)] were used for the statistical analyses.

Primary statistics showed that there was a high variation and poor overlapping in number of observations among seasons and age classes (Table 1). To overcome the statistical problems this raises in the analyses, monofactorial analyses were performed.

**Table 1:** The number of sampled female wild boars in total, per season, per age class, and per sampling region

Season	Total	Age class				Region			
		Juvenile	Yearling	Adult	Unknown	Skåne	Blekinge	Södermanland	Uppland
Spring	76	11	16	8	41	0	5	59	12
Summer	48	7	17	8	16	0	2	44	2
Autumn	345	109	100	101	35	38	149	78	80
Winter	106	23	15	27	41	45	2	31	28
Total	575	150	148	144	133	83	158	212	122

#### Sub-analysis 1

Analyses of the effect of season and age class on the continuous variables weight (dressed), OR and litter size. The statistical model included either the fixed effect of season (4 classes) or age class (3 classes). These analyses was performed using PROC GLM, and were based on 421, 139 and 71 observations, respectively.

#### Sub-analysis 2

For the analyses of the three binary variables, indicating the reproductive stage (anoestrus, cyclic or pregnant), fixed effects of season and age class were included in the model, one at a time. In these analyses prepubertal animals and animals with disrupted cycles were excluded. These analyses were performed using PROC GLIMMIX, and were based on 357 (for season) and 261 (for age class) observations. Using this approach, the effect of age class on the pregnancy rate of female wild boars was also analysed (442 observations). The pregnancy rate was defined as the proportion of pregnant females per age class of the sampled population. P-values less than 0.05 were considered as significant.

## Results

**Fig. 1:**
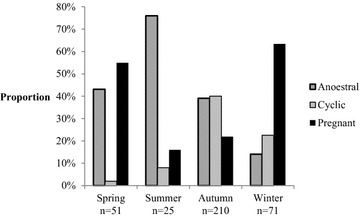
The proportion of female wild boar in different reproductive stages in relation to culling season (prepubertal animals and animals with disrupted ovarian cyclic pattern excluded)

In total, 617 female wild boars were collected and 575 individuals met the requirements to be included in this study, i.e. a complete set of reproductive organs available for examination. The number of animals sampled in each season and in relation to age and sampling region is presented in Table 1. The number of animals in the different reproductive stages were: 207 prepubertal (36.0%), 131 anoestral (22.8%), cyclic 104 (18.1%), 123 pregnant (21.2%), and 11 had a disrupted ovarian cyclic pattern (1.9%). The levels of significance for the effects included in the statistical analyses are presented in Table 2. The proportion of animals in different reproductive stages (prepubertal animals and animals with disrupted oestrus cycle excluded) was significantly affected by season (P < 0.0001, Fig. 1).

**Table 2:** The levels of significance for the effects of season and age class on weight, reproductive potential, reproductive stage and pregnancy rate of the sampled female wild boars

Variable	Season	Age class
Weight	***	***
Reproductive potential		
OR	Ns	***
Litter size	Ns	***
Reproductive stage		
Anoestrus	***	**
Cyclic	***	Ns
Pregnant	***	Ns
Pregnancy rate	–	***

Levels of significance: *ns* not significant

P  < 0.05; ** P ≤ 0.01; *** P ≤ 0.001

**Fig. 2:**
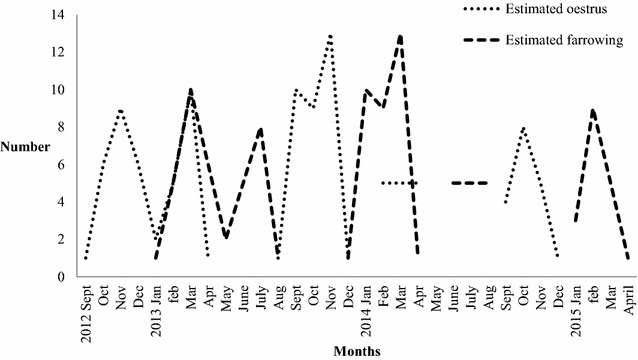
The estimated oestrus/mating—and farrowing months based on the crown-rump-length (CRL) of embryos/fetuses from 105 pregnant wild boars

Pregnancy was detected in 122 animals. After excluding 11 pregnant females with embryo mortality and 10 animals in an early pregnancy state (<2.5 weeks), the mean number of CL was 6.4 (range 2–11; n = 101) and the mean number of embryos/foetuses was 5.4 (range 1–9). The estimated oestrus/mating- and farrowing months based on the CRL of embryos/foetuses from 105 pregnant female wild boars sampled between November 2012 and December 2014, are illustrated in Fig. 2.

**Fig. 3:**
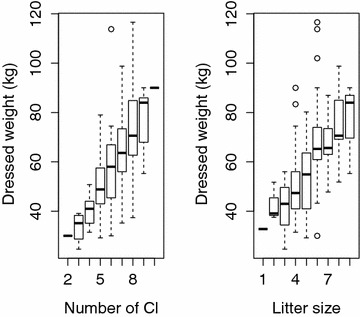
The number of *corpora lutea* (ovulation rate) and litter size in relation to maternal field dressed weight in kg. Box and whisker plots show median (*point within box*), 25% (interquartile range 1) and 75% (interquartile range 3) percentiles (box) and the range (whiskers). *Circles* indicate statistical outliers

Age class and weight was highly associated (Table 2). The reproductive potential (OR and litter size) in relation to weight class for 99 of the pregnant animals, for which the FDW was noted and the pregnancy had proceeded over 18 days, is illustrated in Fig. 3. The reproductive potential increased significantly with age class. Age was determined in 442 animals of which 78 were pregnant, and the pregnancy rate in relation to age class is presented in Table 3. The pregnancy rate increased significantly per age class (P < 0.001).

**Table 3:** Pregnancy rate of the examined female wild boars in relation to age class

Age class	Number examined	Number (%) pregnant
Juvenile	150	10 (6.7)
Yearling	148	30 (20.3)
Adult	144	38 (26.4)
Total	442	78 (17.7)

## Discussion

To identify the population dynamics of a game species in order to conduct a proper management, understanding of its reproductive period, oestrus cycle and the mean reproductive outcome is essential. In the present study, the reproductive pattern and potential among free ranging female wild boars in Sweden is described.

The reproductive potential indicated by ovulation rates and litter size of the studied wild boars was high (6.4 and 5.4, respectively), compared to litter sizes reported from other countries, e.g. Portugal 4.2 [15], Iberian Peninsula 3.6 [26], Switzerland 4.8 [27], and Italy 5.0 [1], but not as high as in a recent German study; 6.6 [8]. The reproductive potential of wild boars can be affected by many factors such as feed availability, climate [6, 26, 28, 29], the genetic background including influence of domestic pigs [14, 30, 31], and the weight and age of gilts and sows [9, 29]. High natural feed availability may increase the litter size [32] as well as the availability of supplementary feed [26, 29]. In Sweden, although controversial, supplemental feeding is to a varying extent applied throughout the wild boar range. This makes studies of the direct effects of supplemental feeding on free ranging wild boars, including controls (in areas without supplementary feeding), more or less impossible to conduct in Sweden. Moreover, the availability of natural feed in Sweden is high for wild boars, both in forests and agricultural habitats, and especially in the southern and central parts of Sweden. During winter, the scarcity of natural feed is compensated by supplemental feeding. Compared to southern and central Europe [12], Swedish summers are (due to mild climate) not likely to constitute a regulating season for wild boar populations. Instead, the summer conditions in the south and central Sweden normally result in a long vegetation period without long periods of high ambient temperatures or drought [22]. Such conditions are favorable for wild boars [8, 28] and possibly contribute to the large litter sizes.

The genetic background and possible influence of domestic pigs in the wild boar population is known to affect the litter size and growth rate [30]. Bergman et al. [33] found genetic signs in Swedish wild boars that possibly could indicate interbreeding between wild boars and domestic pigs. Still, further studies to identify the occurrence of genetic introgression from domestic pig into the Swedish wild boar populations are required for wider conclusions.

The age/weight of the sows in the wild boar population can also affect the observed litter size. Younger and lighter individuals have smaller litters than older/heavier individuals [12, 26]. In agreement with previous studies [1, 8, 15, 26, 34], we also found that pregnancy rate and reproductive potential increased with age and weight. The proportion of old/heavy sows, included in the studied material, is highly linked to management in Sweden. The Swedish hunting regulations [35] prohibit culling of females with piglets, and large females (also without piglets) are often voluntarily banned from culling by hunters. Therefore, it is possible that the age and weight composition of the sampled animals was not representative for the actual age and weight composition of the wild boar population in the sampled areas. Instead, it is likely that the proportion of large, adult females was higher in the population than in the studied material, and the average reproductive potential presented here might be underestimated.

Although the majority of the studied female wild boars showed a seasonal pattern in accordance to previous studies [2, 9], cyclic and pregnant females were found in all seasons. This pattern was further confirmed by the estimated oestrus/mating and farrowing months based on the CRL of embryos/fetuses (Fig. 2). These observations show that farrowing may occur ‘off-season’ in Sweden, as also suggested by studies describing the corresponding pattern when feed is accessible throughout the year [15, 36]. Despite the lack of material from a few months, a tendency of at least two peak periods for oestrus/mating (one in November and one in March) is seen in the material from 2013 and evident, but not as pronounced in 2014 (Fig. 2). This would give (if the animals had stayed alive), peaks of farrowing in March and July. Breeding in March is not considered to be normal for “short-day breeders”, such as wild boars. However, this bimodal distribution of birth has been described in other studies [2, 14]. Other authors explain this second peak as a result of (1) some factors that make an early spring litter die and the sow then goes into oestrus again and become pregnant, (2) involvement of young females in reproduction during spring, (juveniles that did not reach the necessary weight at the time of the mating season), and (3) genetic influences of domestic pigs in the wild boar population that also can affect the seasonality of reproduction [14], a factor that still is to be investigated in the Swedish wild boar population. In the first explanation, harsh weather conditions, scarcity of feed, or diseases are factors that may affect the litter survival. In our case, the female boars involved in the second birth peak (June–July) had a mean live weight of 54.3 kg (n = 13, range 34–70.6 kg), which may contribute to the second theory. Moreover, scarcity of feed would not be an explanation to the second peak because the studied population was supplementary fed throughout the study period. However, during January and February 2013, game managers at the different estates, observed a lot of piglets that later on disappeared. The results of the macroscopical examination of the reproductive tract of female wild boars from this period confirm these observations. We found that a large proportion (17%, n = 84 in 2013) and (26%, n = 27 in 2014) of the females that were shot between January and May (both included), recently had been pregnant. In practice this means that these individuals either (1) had lost their piglets shortly after birth due to piglet death (because of disease, predation, starvation, or frostbite) or the sows had left their piglets of unknown cause; (2) had aborted, i.e. lost their litter before birth due to poor body condition or disease; or (3) were shot although their piglets were alive (unlikely when hunters neither seen piglets or quoted drawn teats of sows). Harsh weather conditions and infectious diseases may contribute to the annual loss of wild boar litters. Still, the appearance and possible effect of infectious diseases on wild boar reproduction, i.e. porcine parvovirus and porcine circovirus type 2 is still to be investigated.

Litters produced all year round complicate wild boar hunting and may thus infer difficulties to meet management goals. Adult sows which, if targeted by hunting, would be the animal category most effective to harvest if a population decrease is aimed for, are protected from culling if accompanied by piglets. Further, piglets present during hunting season, may be injured and/or killed by hunting dogs, which is an important issue of ethical and animal welfare to acknowledge.

## Conclusions

This descriptive study of wild boar reproduction showed that the reproductive potential in the studied population was high and that farrowing may occur ‘off-season’. We suggest that the general high feed availability and environmental conditions in Sweden is favorable for wild boars. The possible effect of introgression of domestic pigs should not be neglected as a possible contributing factor to the high reproductive potential presented in this study.

## Additional files

**Additional file 1.** Map of Sweden highlighting the regions where female wild boars were sampled. a) Skåne, b) Blekinge c) Södermanland, d) Uppland.

**Additional file 2.** Mean temperature and percipitation in four Swedish counties, measured in January and July in the years of 2013–2015.

## References

[1] Boitani L, Trapanese P, Mattei L (1995). Demographic patterns of a wild boar (*Sus scrofa* L.) population in Tuscany, Italy. IBEX J Mount Ecol..

[2] Ježek M, Štípek K, Kušta T, Červený J, Vícha J (2011). Reproductive and morphometric characteristics of wild boar (*Sus scrofa*) in the Czech Republic. J For Sci..

[3] Keuling O, Baubet E, Duscher A, Ebert C, Fisher C, Monaco A (2013). Mortality rates of wild boar *Sus scrofa* L. in central Europe. Eur J Wildl Res.

[4] Massei G, Kindberg J, Licoppe A, Cačić D, Šprem N, Kamler J, Baubet E (2015). Wild boar populations up, numbers of hunters down? A review of trends and implications for Europe. Pest Manag Sci.

[5] Svenska Jägareförbundet [The Swedish Hunting Association]. 2014. http://svenskjakt.se/Start/Nyheter/2014/08/hur-mycket-vilt-har-vi-egentligen/. Accessed 19 July 2016.

[6] Servanty S, Gaillard JM, Allaine D, Brandt D, Baubet E (2007). Litter size and fetal sex ratio adjustment in a highly polytocous species: the wild boar. Behav Ecol.

[7] Malmsten A, Jansson G, Dalin AM (2017). Postmortem examination of the reproductive organs of female wild boars (*Sus scrofa*) in Sweden. Reprod Dom Anim..

[8] Frauendorf M, Gethöffer F, Siebert U, Keuling O (2016). The influence of environmental and physiological factors on the litter size of wild boar (*Sus scrofa*) in an agriculture dominated area in Germany. Sci Total Environ.

[9] Mauget R, Cole DJA, Foxcroft GR (1982). Seasonality of reproduction in the wild boar. Control of pig reproduction.

[10] Pépin D, Spitz F, Janeau G, Valet G (1987). Dynamics of reproduction and development of weight in the wild boar (*Sus scrofa*) in South-West France. Z Säugetierk..

[11] Delcroix I, Mauget R, Signoret JP (1990). Existence of synchronization of reproduction at the level of the social group of the European wild boar (*Sus scrofa*). J Reprod Fert..

[12] Mauget R, Assenmacher I, Farner DS (1978). Seasonal reproductive activity of the European wild boar. Comparison with the domestic sow. Environmental endocrinology.

[13] Macchi E, Starvaggi Cucuzza A, Badino P, Odore R, Re F, Bevilacqua L, Malfatti A (2010). Seasonality of reproduction in wild boar (*Sus scrofa*) were assessed by fecal and plasmatic steroids. Theriogenology.

[14] Mauget R (1972). Observations sur la reproduction du sanglier (*Sus scrofa* L.) a l’état sauvage. Ann Biol Anim Biochem Biophys..

[15] Fonseca C, Santos P, Monzón A, Bento P, Alves da Silva A, Alves J (2004). Reproduction in the wild boar (*Sus scrofa* Linnaeus, 1758) populations of Portugal. Galemys..

[16] Orlowska L, Rembacz W, Florek C (2012). Carcass weight, condition and reproduction of wild boar harvested in north-western Poland. Pest Manage Sci..

[17] Lemel J, Truvé J, Söderberg B (2003). Variation in ranging and activity behavior of European wild boar *Sus scrofa* in Sweden. Wildl Biol.

[18] Truvé J, Lemel J (2003). Timing and distance of natal dispersal for wild boar *Sus scrofa* in Sweden. Wildl Biol..

[19] Thurfjell H, Ball JP, Åhlén PA, Kornacher P, Dettki H, Sjöberg K (2009). Habitat use and spatial patterns of wild boar *Sus scrofa*: agricultural fields and edges. Eur J Wildl Res.

[20] Welander J (2000). Spatial and temporal dynamics of wild boar (*Sus scrofa*) rooting in a mosaic landscape. J Zool.

[21] Malmsten A, Dalin AM (2016). Puberty in female wild boar (*Sus scrofa*) in Sweden. Acta Vet Scand.

[22] SMHI.se. 2016. Vegetationperiod. http://www.smhi.se/kunskapsbanken/klimat/vegetationsperiod-1.6270. Accessed 30 June 2016.

[23] Naturvårdsverket. 2016. http://www.naturvardsverket.se/Var-natur/Jakt/Jakt-pa-klovvilt/Jakt-pa-vildsvin/. Accessed 13 Sept 2016.

[24] Matschke GH (1967). Ageing European wild hogs by dentition. J Wildl Manag..

[25] Henry VG (1968). Fetal development in European wild hogs. J Wildl Manag..

[26] Fernández-Llario P, Mateos-Quesada P (1998). Body size and reproductive parameters in the wild boar *Sus scrofa*. Acta Theriol..

[27] Moretti M (1995). Birth distribution, structure and dynamics of a hunted mountain population of wild boars (*Sus scrofa* L.), Ticino, Switzerland. IBEX J Mount Ecol..

[28] Geisser H, Reyer HU (2005). The influence of food and temperature on population density of wild boar *Sus scrofa* in the Thurgau (Switzerland). J Zool.

[29] Fernández-Llario P, Carranza J (2000). Reproductive performance of the wild boar in a Mediterranean ecosystem under drought conditions. Ethol Ecol Evol..

[30] Booth WD (1995). Wild boar farming in the United Kingdom. IBEX J Mount Ecol..

[31] Gongora J, Peltoniemi OAT, Tammen I, Raadsma H, Moran C (2003). Analyses of possible domestic pig contribution in two populations of finnish farmed wild boar. Acta Agric Scand.

[32] Massei G, Genov PV, Staines BW (1996). Diet, food availability and reproduction of wild boar in a Mediterranean coastal area. Acta Theriol..

[33] Bergman IM, Sandholm K, Nilsson Ekdahl K, Okumura N, Uenishi H, Guldbrandtsen B (2012). MBL1 genotypes in wild boar populations from Sweden, Austria, the Czech Republic, and Japan. Int J Immunogenet.

[34] Santos P, Fernández-Llario P, Fonseca C, Monzón A, Bento P, Soares AMVM (2006). Habitat and reproductive phenology of wild boar (*Sus scrofa*) in the western Iberian Peninsula. Eur J Wildl Res.

[35] Jaktförordning. 1987:905. https://www.notisum.se/rnp/sls/lag/19870905.htm#B1. Accessed 2 Feb 2017.

[36] Bieber C, Ruf T (2005). Population dynamics in wild boar *Sus scrofa*: ecology, elasticity of growth rate and implications for management of pulsed resource consumers. J Appl Ecol.

