# Supplementary material for: The reproductive pattern and potential of free ranging female wild boars (Sus scrofa) in Sweden
Source: Acta Vet Scand. 2017 Aug 1;59:52. doi: 10.1186/s13028-017-0321-0 (PMC5539618; doi:10.1186/s13028-017-0321-0)
Supplement: Supplementary file 1 — Additional file 1. Map of Sweden highlighting the regions where female wild boars were sampled. a) Skåne, b) Blekinge c) Södermanland, d) Uppland. [file 13028_2017_321_MOESM1_ESM.pdf]

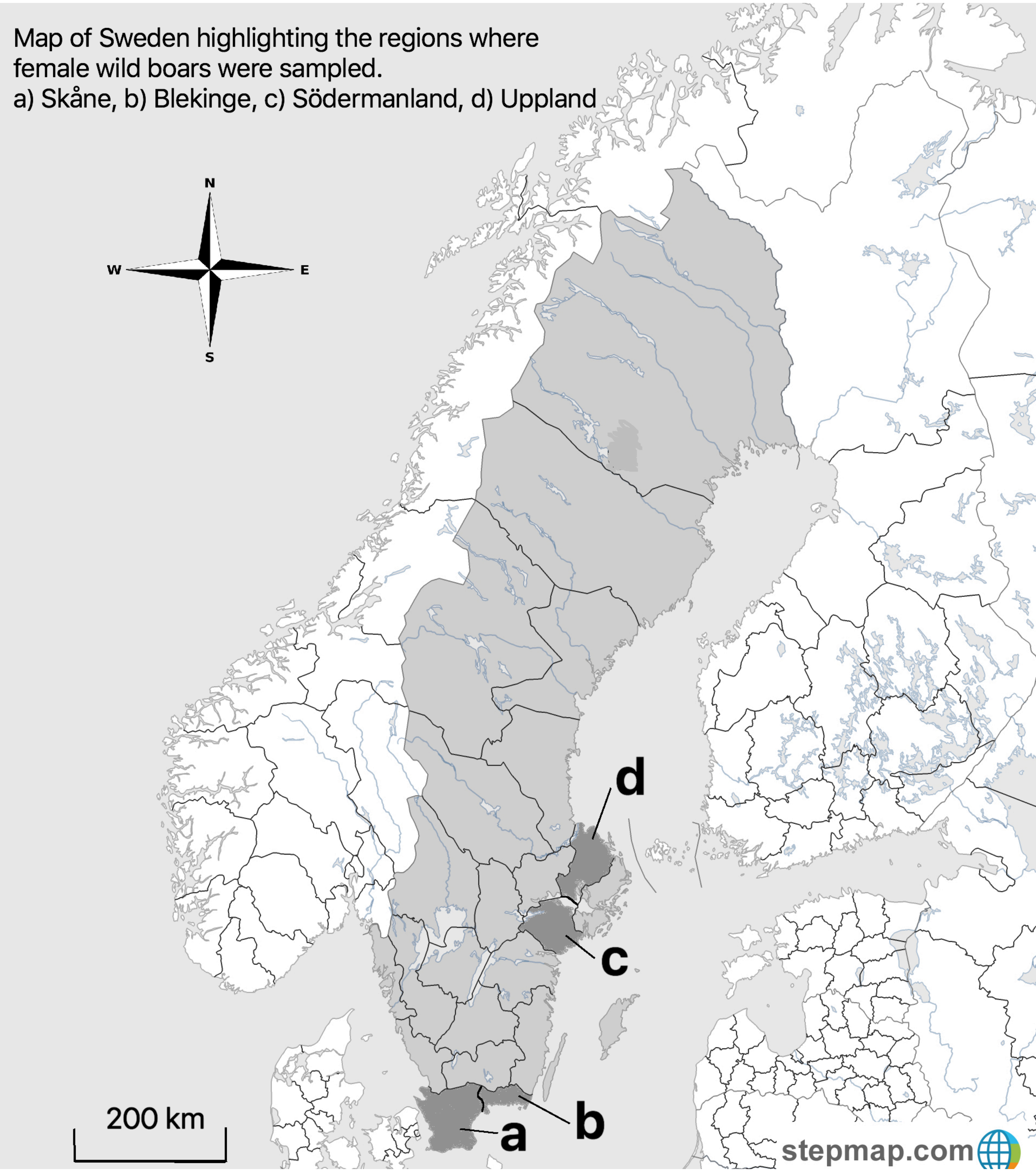

Map of Sweden highlighting the regions where female wild boars were sampled.
a) Skåne, b) Blekinge, c) Södermanland, d) Uppland
