# Supplementary material for: The reproductive pattern and potential of free ranging female wild boars (Sus scrofa) in Sweden
Source: Acta Vet Scand. 2017 Aug 1;59:52. doi: 10.1186/s13028-017-0321-0 (PMC5539618; doi:10.1186/s13028-017-0321-0)
Supplement: Supplementary file 2 — Additional file 2. Mean temperature and percipitation in four Swedish counties, measured in January and July in the years of 2013–2015. [file 13028_2017_321_MOESM2_ESM.docx]

|  | **Skåne** | **Blekinge** | **Södermanland** | **Uppsala** |
| --- | --- | --- | --- | --- |
| **January 2013** |  |  |  |  |
| Mean temperature (Cº) | -2.0 | -2.0 | -5.9 | -5.0 |
| Mean percipitation (mm) | 39.6 | 24.3 | 24.6 | 25.4 |
| **July 2013** |  |  |  |  |
| Mean temperature (Cº) | 18.1 | 17.8 | 16.7 | 17.6 |
| Mean percipitation (mm) | 63.8 | 58.5 | 42.4 | 17.1 |
| **January 2014** |  |  |  |  |
| Mean temperature (Cº) | 1.0 | 0.1 | -2.2 | -2.8 |
| Mean percipitation (mm) | 52.5 | 44.7 | 44.4 | 43.8 |
| **July 2014** |  |  |  |  |
| Mean temperature (Cº) | 19.5 | 19.5 | 19.3 | 19.8 |
| Mean percipitation (mm) | 84.2 | 69.9 | 22.9 | 41.6 |
| **January 2015** |  |  |  |  |
| Mean temperature (Cº) | 2.1 | 1.9 | 0.4 | -0.4 |
| Mean percipitation (mm) | 84.2 | 43.9 | 80.8 | 85.1 |
| **July 2015** |  |  |  |  |
| Mean temperature (Cº) | 16.2 | 16.0 | 15.8 | 16.7 |
| Mean percipitation (mm) | 46.0 | 50.5 | 87.6 | 112.9 |

**Additional file 2** Mean temperature and percipitation in four Swedish counties, measured in January and July in the years of 2013-2015.
